# Identification of genes related to high royal jelly production in the honey bee (*Apis mellifera*) using microarray analysis

**DOI:** 10.1590/1678-4685-GMB-2017-0013

**Published:** 2017-10-02

**Authors:** Hongyi Nie, Xiaoyan Liu, Jiao Pan, Wenfeng Li, Zhiguo Li, Shaowu Zhang, Shenglu Chen, Xiaoqing Miao, Nenggan Zheng, Songkun Su

**Affiliations:** 1 College of Bee Science, Fujian Agriculture and Forestry University, Fuzhou, China; 2 College of Animal Sciences, Zhejiang University, Hangzhou, China; 3 Research School of Biology, College of Medicine, Biology and Environment, The Australian National University, Canberra, Australia; 4 Qiushi Academy for Advanced Studies, Zhejiang University, Hangzhou, China

**Keywords:** honeybee, royal jelly production, gene chip, molecular marker, differentially expressed genes

## Abstract

China is the largest royal jelly producer and exporter in the world, and high royal jelly-yielding strains have been bred in the country for approximately three decades. However, information on the molecular mechanism underlying high royal jelly production is scarce. Here, a cDNA microarray was used to screen and identify differentially expressed genes (DEGs) to obtain an overview on the changes in gene expression levels between high and low royal jelly producing bees. We developed a honey bee gene chip that covered 11,689 genes, and this chip was hybridised with cDNA generated from RNA isolated from heads of nursing bees. A total of 369 DEGs were identified between high and low royal jelly producing bees. Amongst these DEGs, 201 (54.47%) genes were up-regulated, whereas 168 (45.53%) were down-regulated in high royal jelly-yielding bees. Gene ontology (GO) analyses showed that they are mainly involved in four key biological processes, and pathway analyses revealed that they belong to a total of 46 biological pathways. These results provide a genetic basis for further studies on the molecular mechanisms involved in high royal jelly production.

## Introduction

The honey bee (*Apis mellifera*) is an important insect that generates high economic and ecological values for humans as a key pollinator of crops (Morse and Calderone, 2000; Klein *et al.*, 2007) and producer of bee products, including honey, royal jelly (RJ), pollen, propolis and beeswax (Schmidt, 1997). A published genome (The Honey Bee Genome Sequencing Consortium, 2006) and its biological characteristics have also contributed to the recent emergence of honey bees as a new model organism for research in diverse areas of learning and memory (Zhang *et al.*, 2006; Adler, 2013), division of labour (Ament *et al.*, 2008, 2010), caste differentiation (Barchuk *et al.*, 2007; Li *et al.*, 2013), genetics (Page Jr *et al.*, 2012) and breeding (Hyink *et al.*, 2013).

Royal jelly is the principal food consumed by larval and adult honey bee queens and is secreted by the hypopharyngeal and mandibular glands of nursing worker bees. Royal jelly is rich in various nutrients beneficial to humans, including proteins, sugars, vitamins and a large number of bioactive substances, such as 10-hydroxy-2-decenoic acid (Viuda-Martos *et al.*, 2008). Royal jelly is used as a health food and natural cosmetic in many countries (Ramadan and Al-Ghamdi, 2012). The market value of royal jelly is considerably higher than that of honey or pollen (Bogdanov, 2011; Ramadan and Al-Ghamdi, 2012), and royal jelly production has become a major income source of many beekeepers in China.

China is the largest royal jelly producer and exporter in the world, with current exports accounting for more than 90% of the total international trade of royal jelly, and this value resulted from the development, establishment and refinement of a high royal jelly-producing strain of the honey bee (*Apis mellifera ligustica*) (Cao *et al.*, 2016). Zhenongda No. 1 is a honey bee strain with higher production of honey and royal jelly than wild-type *Apis mellifera ligustica* (Chen *et al.*, 2002). This strain is well known for its genetic differences and royal jelly production ability. Previous studies have identified the genetic markers of high royal jelly-producing bees, including morphological (Su and Chen, 2003), cytological markers (Chen *et al.*, 2005) and biochemical markers (Zhang *et al.*, 2007; Li *et al.*, 2008; Jianke *et al.*, 2010; Zheng *et al.*, 2010).

However, the molecular mechanisms underlying genetic differences in the ability of honey bees to produce royal jelly remain unknown. Microarray chips are a high throughput and mass data processing technology used to analyse differentially expressed genes (DEGs) in specific biological processes. Microarrays have been extensively used in the study of honey bees, including age-related division of labour (Kucharski and Maleszka, 2002; Grozinger *et al.*, 2003; Whitfield *et al.*, 2003), caste differentiation (Barchuk *et al.*, 2007), susceptibility to *Varroa* parasitism (Navajas *et al.*, 2008), as well as immunity and disease (Evans, 2006). Dozens of crucial genes can be screened using DNA microarrays for further functional identification. In the present study, 369 DEGs were identified contrasting high royal jelly producing bees and low royal jelly producing bees using chip analysis. This study is the first to conduct a large-scale analysis on gene expression differences between high royal jelly producing bees and low royal jelly producing bees. The results provide a broad perspective on the genes involved in royal jelly production, thereby providing further insights into the mechanism of the royal jelly-producing trait.

## Material and Methods

### Honey bee colonies and sample collection

High royal jelly-producing colonies (Zhenongda No. 1), which were derived from an Italian honey bee subspecies (*Apis mellifera ligustica*), were obtained from the experimental apiary of Zhejiang University, Hangzhou, China. Local Italian bees with a low royal jelly-producing trait were obtained from Miao Siwei Apiary, Simao County, Yunnan Province.

To reduce differences in their genetic background between high and low royal jelly producing colonies, the virgin queens of the low royal jelly-yielding strain were delivered to the experimental apiary of Zhejiang University and there mated naturally with local drones of the high royal jelly-yielding strain to generate F1 progeny. Then, virgin queens were raised from F1 offspring and back-crossed to drones of the high royal jelly-yielding strain. The colonies of high royal jelly producing bees and low royal jelly producing bees were derived from the back-cross progeny colonies, and the royal jelly production of the back-cross progeny colonies was measured prior to sample collection. The collection method of royal jelly was performed as described in a previous report, with minor modifications (Jianke *et al.*, 2010). Each colony was provided with two plastic strips harbouring 128 queen cell cups with grafted one-day-old larvae, and royal jelly was collect after 72 h of larval grafting. Royal jelly collections were done three times for each colony, and the samples were weighed with a digital scale (Mettler Toledo, Colombus, OH, USA; accurate to 0.001 g). Four colonies of high royal jelly producing bees with a high royal jelly production and four colonies of low royal jelly producing bees with a low royal jelly production were selected from the back-cross progeny colonies and then used as material for chip and qRT-PCR analyses. The nurses were caught at the time when they entered the queen cell cups and were feeding the larvae. All of the collected bees were frozen immediately in liquid nitrogen and then stored at −80 °C until analysis.

### Microarray construction

A honey bee chip was designed following the Agilent eArray Design guidelines. It contained 11,689 genes from GenBank (http://www.ncbi.nlm.nih.gov/gene/?term=honey bee) and gene sequences obtained from our lab sequence results, not yet deposited in NCBI. Each gene had a 60 bp sequence included in the oligonucleotide microarray construction done by Advanced Throughput Inc. (Shanghai, China). The details of probes are provided as Supplementary material (Table S1).

### RNA extraction, One-Color labelling and microarray hybridisation

Heads of nursing bees collected from the honey bee samples were cleaned in PBS solution to remove other tissues. Total RNA was extracted from pools of 30 heads per colony using TRIzol (Invitrogen, Waltham, MA, USA) according to the procedures of a previous study (Liu *et al.*, 2011). RNA concentration and quality were assessed using a NanoDrop 2000 spectrophotometer (NanoDrop 2000, Thermo Fisher Scientific, Waltham, MA, USA) and an Agilent 2100 Bioanalyzer, respectively, and were replicated for four colonies each for high royal jelly producing bees and low royal jelly producing bees.

The One-Color Quick Amp Labelling Kit (Agilent, Santa Clara, CA, USA) was used to generate fluorescent cRNA according to the manufacturer's instructions. Aliquots of 200 ng of total RNA from each sample were used in a protocol described by Ferrari *et al.* (2011), and 1.5 μg of Cy3-labelled cRNAs were obtained from each sample. The cRNAs were fragmented for 15 min and hybridised for 17 h at 65 °C in 2GE hybridisation buffer HI-RPM following the instruction of Agilent Gene Expression Hybridization Kit (Agilent).

### Chip scanning and data analysis

After hybridisation, the slides were disassembled using GE wash buffer 1 and washed twice with GE wash buffers 1 and 2 for 1 min at room temperature. The chip was then scanned with an Agilent scanner. Raw data were obtained and data normalisation was conducted using the Agilent Feature Extraction Software (Zahurak *et al.*, 2007). Standardized data were provided as Table S2. DEGs with at least twofold changes between high royal jelly producing bees and low royal jelly producing bees were screened for analysis (log_2_ ratio > 1; ratio: the fluorescence intensity of high royal jelly producing bees/low royal jelly producing bees) following methods described previously (Gerhold *et al.*, 2001). Cluster analysis was performed using Cluster 3.0 and TreeView software.

### Bioinformatics analysis

GO and pathway analyses were performed to track the functional annotation and biological pathways of DEGs using ClueGo (Bindea *et al.*, 2009) referencing the DroSpeGe database (Gilbert, 2007) and KEGG.

### qRT-PCR assays of the selected genes

Total RNA extraction and cDNA synthesis were performed as described previously (Nie *et al.*, 2014). The primers, which were designed using Primer Premier 5.0, are listed in Table S3. qRT-PCR in our study was performed using the LineGeneK PCR System (Bioer Technology Co., Ltd., Hangzhou, China). The PCR mix (20 μL total) consisted of 2 μL of 250 ng/μL cDNA template, 10 μL THUNDERBIRD SYBR qPCR Mix (QPS-201, TOYBO, Osaka, Japan), 1 μL of 10 μM forward primer, 1 μL of 10 μM reverse primer and 6 μL of nuclease-free water. The reactions were run as follows: 95 °C for 1 min, 40 cycles of 15 s at 95 °C and 45 s at 60 °C. GAPDH (forward: GATGCACCCATGTTTGTTTG; reverse: TTTGCAGAAGGTGCATCAAC) was used as an internal control gene because of its stable expression level in the heads of honey bees (Scharlaken *et al.*, 2008). All reactions were run in triplicates. The Ct values were analysed using default threshold settings, and the mean Ct values of each triplicate sample were used for the subsequent analysis. The relative quantification of the selected gene expression was calculated using 2^−ΔΔCt^ method (Livak and Schmittgen, 2001).

### Statistical analysis

Data analysis of the royal jelly yield between high royal jelly producing bees and low royal jelly producing bees used for gene chip and qRT-PCR was performed using the Data Processing System (DPS) software (Tang and Zhang, 2013). Independent-sample *t* tests implemented in SPSS 13.0 software were performed to analyse the results.

## Results

### DEGs between high royal jelly producing bees and low royal jelly producing bees

**Figure 1:**
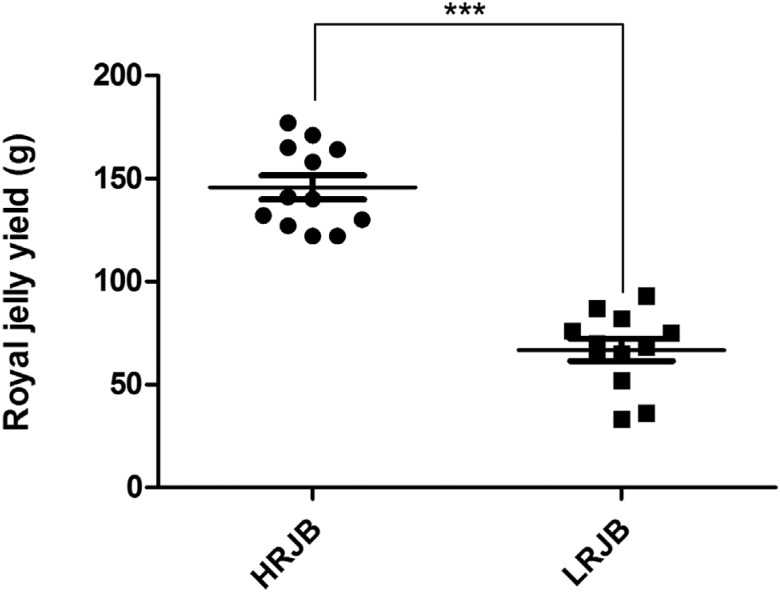
Royal jelly yield of high royal jelly producing bees (HRJB) and low royal jelly producing bees (LRJB). Four colonies of high royal jelly producing bees and four colonies of low royal jelly producing bees were selected from the back-cross progeny colonies. The royal jelly collections were performed three times for each colony and weighed with a digital scale. Independent-sample *t*-tests were performed to analyse the results using the SPSS 13.0 software. *p < 0.001)

**Figure 2:**
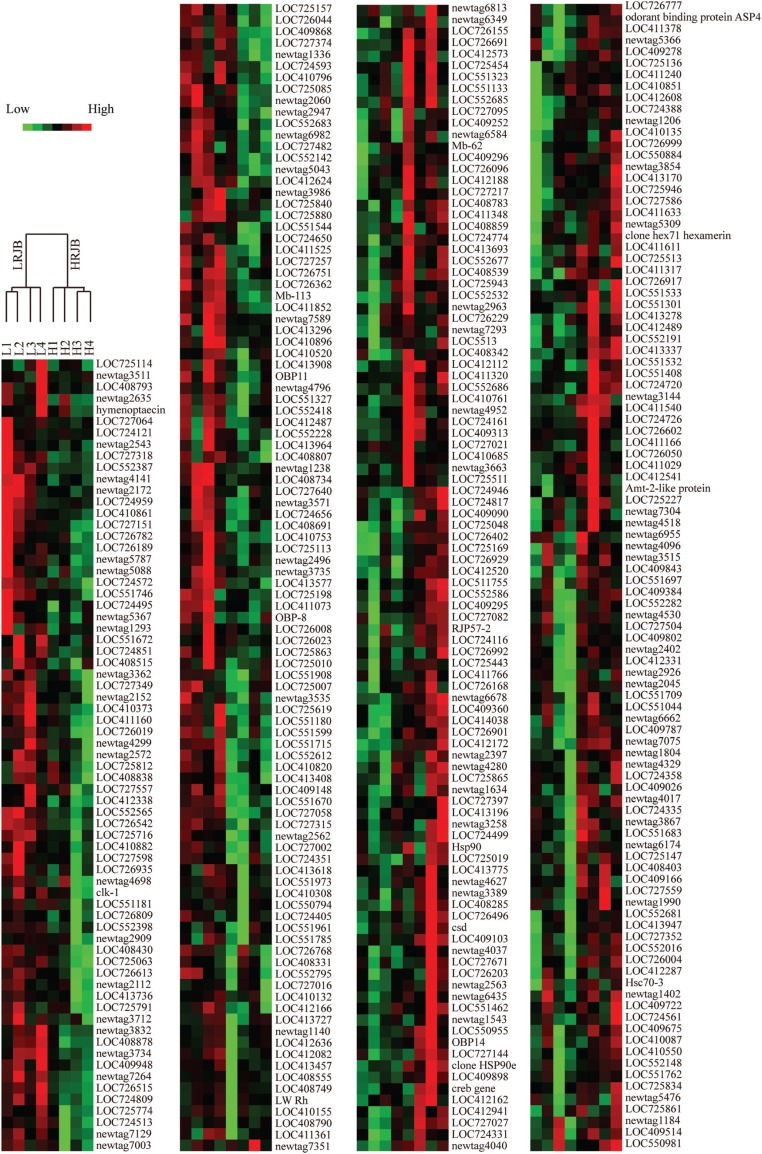
Hierachical clustering analysis of 369 differently expressed genes. Each horizontal row indicates a gene, and each vertical column represents a colony. In the top of the figure, the tree diagram represents the eight colony samples, which are divided into two groups: high royal jelly producing bees (HRJB) and low royal jelly producing bees (LRJB). Red represents up-regulated genes in HRJB; green represents down-regulated genes in HRJB. Light shades reflect different levels of up- or down-regulated genes.

Four colonies of high royal jelly producing bees and four colonies of low royal jelly producing bees were selected from the back-cross progeny colonies. Royal jelly production of high royal jelly producing bees was 122 g to 177 g with an average of 145.75 g. The royal jelly production of low royal jelly producing bees was 33 g to 93 g with an average of 66.75 g. Production of royal jelly between high royal jelly producing bees and low royal jelly producing bees exhibited a significant difference (Figure 1). This finding indicated that the colonies were reliable for the subsequent study. After hybridisation, scanning and data analysis, 369 DEGs between high royal jelly producing bees and low royal jelly producing bees with at least twofold changes were identified (Table S4). Amongst these modulated genes, 201 (54.47%) were up-regulated, whereas 168 (45.53%) were down-regulated in high royal jelly producing bees. Hierarchical clustering analysis showed that four high royal jelly bee colonies were clustered, and the other four low royal jelly bee colonies were clustered, indicating that the samples used for microarray are reliable (Figure 2).

### Gene ontology (GO) and Kyoto Encyclopaedia of Genes and Genomes (KEGG) pathway analyses

**Figure 3:**
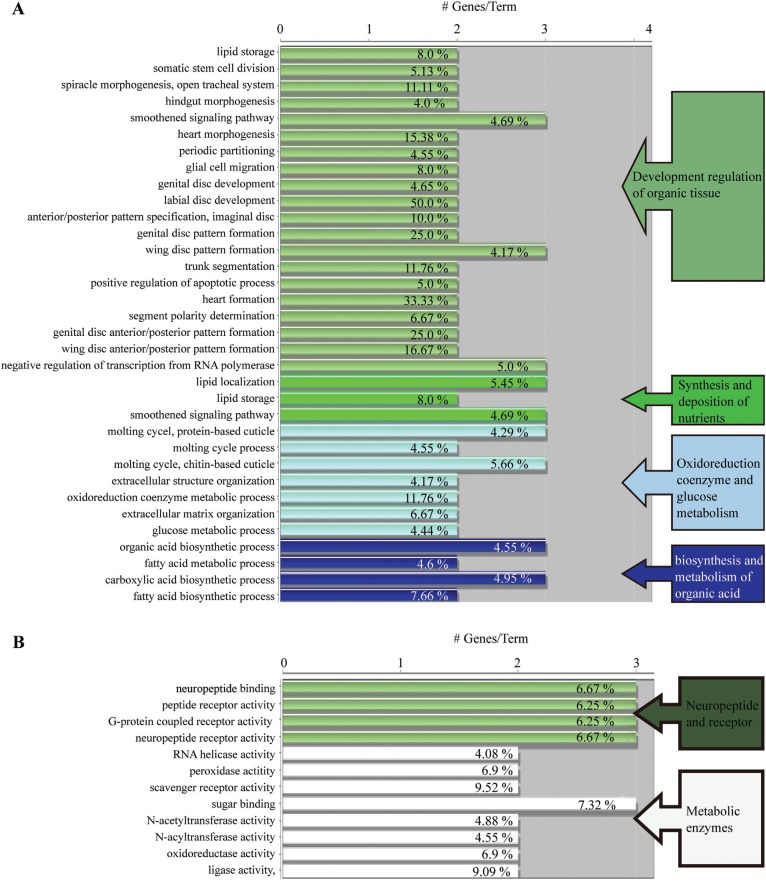
Analysis on functional enrichment of the differently expressed genes in the high royal jelly producing bees (HRJB) and low royal jelly producing bees (LRJB). Gene ontology (GO) pathway terms specific for differently expressed genes. The bars represent the number of genes associated with the terms. The percentage of genes per term is shown as a bar label. (A) Biological process enrichments of the identified genes. The gene functions are listed on the left and the biological process in which these genes are involved is shown on the right. (B) Molecular function enrichments of the identified genes.

GO analysis was used to classify possible functions of genes. Based on sequence homology, these functions were broadly categorised into four groups according to biological process, namely, developmental regulation of organic tissue, synthesis and deposition of nutrients, oxidoreduction coenzyme and glucose metabolism, and biosynthesis and metabolism of organic acid (Figure 3A). According to molecular functions, these were divided into two categories, namely, neuropeptide receptor activity and metabolic enzyme activity. The two categories were further broken down into 12 detailed groups: neuropeptide binding, peptide receptor activity, G protein-coupled receptor activity (GPCR), neuropeptide receptor activity, RNA helicase activity, peroxidase activity, scavenger receptor activity, sugar binding, *N*-acetyl transferase activity, *N*-acyl transferase activity, oxidoreductase activity, ligase activity (Figure 3B).

We mapped DEGs to the reference canonical pathways in KEGG to identify the biological pathways that participate in royal jelly production. They were involved in 46 KEGG metabolic pathways, including 4 signalling pathways (*i.e.*, Wnt signalling pathway, hedgehog signalling pathway, TGF-beta signalling pathway and neuroactive ligand–receptor interaction pathway), 35 nutrient anabolic pathways and seven protein processes and modifications (Table S5).

### Validation with quantitative real-time polymerase chain reaction (qRT-PCR)

**Figure 4:**
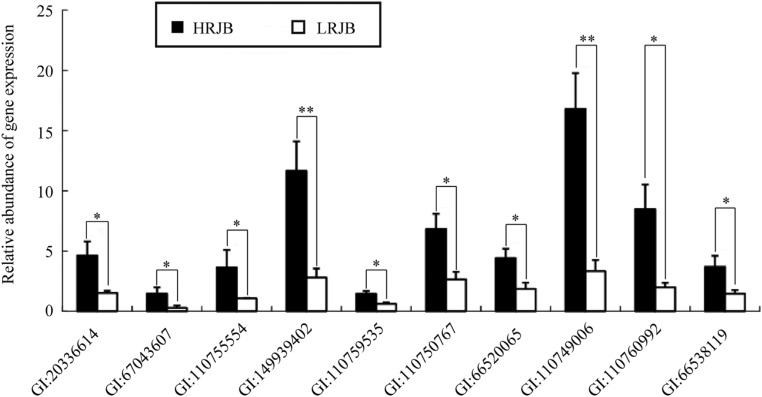
Validation of the differential expression of ten genes in high royal jelly producing bees (HRJB) and low royal jelly producing bees (LRJB) by qRT-PCR analysis. Four colonies of HRJB with a high royal jelly production and four colonies of LRJB with a low royal jelly production were selected from the back-cross progeny colonies, of which heads of nursing bees (n = 30) were collected per colony were used as materials for qRT-PCR analyses. Independent-sample *t*-tests were performed to analyse the results using the SPSS 13.0 software. *p < 0.05; **p < 0.01)

Ten genes were randomly selected for confirmation using qRT-PCR to verify the accuracy of the identification of the DEG in the chip data. The 10 genes were significantly up-regulated in high royal jelly producing bees via qRT-PCR analysis (Figure 4), which was consistent with chip data expression profiling analysis. This observation indicated the reliability of our chip expression profiling analysis. In NCBI, these 10 genes were annotated as follows: dopamine receptor type D2 (Dop2) (GI: 20336614); Amt-2-like protein (GI: 67043607); similar to CG8862-PA (LOC551715) (GI: 110755554); clone hex71 hexamerin (*hex71*) (GI: 149939402); hypothetical protein LOC726515 (LOC726515) (GI: 110759535); similar to lethal (1) G0168 CG33206-PA, isoform A (LOC411348) (GI: 110750767); similar to SHC-adaptor protein CG3715-PA (LOC412172) (GI: 66520065); similar to CG1998-PA, transcript variant 1 (LOC409360) (GI: 110749006); similar to LDLa domain containing chitin binding protein 1 CG8756-PD, isoform D, transcript variant 1 (LOC551323) (GI: 110760992) and similar to multidrug resistance-associated protein 5 (LOC413947) (GI: 66538119). As they were significantly up-regulated in high royal jelly producing bees, these genes could play an important role in royal jelly production of *Apis mellifera*.

## Discussion

In this study, 369 DEGs were identified between high royal jelly producing bees and low royal jelly producing bees using chip analysis. The KEGG pathway of 201 up-regulated genes in the high royal jelly producing bees were involved in protein synthesis (ribosome, proteasome, aminoacyl-tRNA biosynthesis), amino acid metabolism (glycine, serine and threonine metabolism, arginine and proline metabolism, cysteine and methionine metabolism), nucleotide and fatty acid metabolism (purine metabolism, pyrimidine metabolism, fatty acid metabolism), sugar metabolism (galactose metabolism, amino sugar and nucleotide sugar metabolism), signal transduction (hedgehog signaling pathway, phosphatidylinositol signaling system), oxidation (oxidative phosphorylation, peroxisome), transporter (ABC transporters). This suggests that repeated selection pressure for high royal jelly production has affected different pathways.

Compared to the proteome data on hypopharyngeal gland development comparing Italian and royal jelly producing worker honeybees (Jianke *et al.*, 2010), we identified the same three genes: major royal jelly protein 4, 60 kDa heat shock protein and heat shock 70 kDa protein cognate 3; and some similar genes, including ribosomal protein (similar to ribosomal protein L5, ribosomal protein L40, 40S ribosomal protein S29-like), skeleton (actin-related protein 2), and proteasome (proteasome 25kDa subunit) in this microarray. Major royal jelly protein 4 is a major protein for total royal jelly (Schmitzová *et al.*, 1998; Albert *et al.*, 1999), and it was found up-regulated in high royal jelly bees at the transcriptional and proteomic levels, indicating that the content of major royal jelly protein might be increased. Furthermore, the data for most of the ribosomal proteins, heat shock proteins and proteasome found increased in high royal jelly bees, were also consistent with previous studies (Mao *et al.*, 2009; Jianke *et al.*, 2010; Ji *et al.*, 2014), suggesting that these genes might accelerate protein biosynthesis during nursing behaviour. Interestingly, odorant binding protein 4 (OBP4), OBP14 and odorant receptor 22 were up-regulated expressed in the high royal jelly bees, indicating that these genes may easily perceive some chemical signals to make worker manifest nursing behaviours.

Storage protein *hex71*, also called *hex70a*, belongs to the hexamerin family. Hexamerins are synthesised in fat body cells and are secreted into the hemolymph where they accumulate (Telfer and Kunkel, 1991). *hex71* is involved in nutrient uptake and storage (Braun and Wyatt, 1996). *hex71* expression is increased after adult emergence, maintains a high level of transcripts in adult workers (1 to 15 days), and is decreased when nurse bees become forage bees (18 to 28 days old) (Martins *et al.*, 2008). This pattern approximately coincides with the timing of the nursing behaviour. This observation indicated that *hex71* may be closely related to the feeding behaviour of adult worker bees. However, Hex71 protein cannot be used as an amino acid resource for hypopharyngeal gland activity and royal jelly production because this gene is not expressed in the hypopharyngeal gland tissue of honey bees (Martins *et al.*, 2008). Previous studies showed that nutrient metabolism and storage conditions may have a strong effect on the transformation of nurse bees into forager bees (Toth *et al.*, 2005). *hex71* expression may be closely related to fat body metabolic activity and may play an important role in the physiological development of adult worker bees (Martins *et al.*, 2010). Therefore, we hypothesise that the *hex71* gene is indirectly involved in the synthesis and secretion of royal jelly, but increases the royal jelly yield of a colony by postponing the transition of nurse bees to foraging bees.

Dopamine is a neurotransmitter involved in the regulation of various physiological processes of the central nervous system of many organisms (Schultz, 1992). Dopamine can regulate the motion, circadian rhythm, growth and development, sexual behaviour, endocrine system and cognitive behaviour of insects (Wise, 2004). In honey bees, dopamine production is closely correlated with learning and memory, as well as with movement (Vergoz *et al.*, 2007; Nomura *et al.*, 2009). Dopamine works through membrane receptors (dopamine receptors). In our study, *dop2* was found up-regulated in the high royal jelly producing bees. Previous studies indicated that dopamine regulates the development of honey bee antennal neurons (Perk and Mercer, 2006). We hypothesise that *dop2* is associated with dopamine promoting the development of honey bee antennal neurons. These neurons enable worker bees to find and feed larvae fast and efficiently in the queen cells, thereby increasing the feeding rate and quantity of royal jelly deposited in queen cells, and hence, could ultimately affect the royal jelly yield of the entire colony.

Amt-2-like protein belongs to members of the conserved ammonium transporter (Amt) family. Ammonia transport across biological membranes is a critical feature of nitrogen metabolism, and Amt plays an important role in olfactory signalling (Menuz *et al.*, 2014). In the present study, Amt-2-like protein (GI: 67043607) was up-regulated in the high royal jelly producing bees. Similarly, an aminomethyltransferase (GI: 66523499) gene that participates in the nitrogen metabolism pathway, was also up-regulated in the high royal jelly producing bees. We hypothesise that Amt-2-like may affect olfactory signalling via ammonia transport. Our data indicate that Amt-2-like protein has a critical role in royal jelly production.

In addition to the aforementioned three genes, heat shock protein 90 (HSP90, GI: 229892247) and HSP60 (GI: 110763844) were also up-regulated in the high royal jelly producing bees. HSPs function as binding proteins and molecular chaperones and assist in the folding and processing of new proteins. Several heat shock proteins are expressed in the early development of hypopharyngeal glands in adult worker honey bees, and these proteins reach their peak expression levels between the age of 6 and 12 days (Feng *et al.*, 2009). Hypopharyngeal glands, which contain secretory cells in large quantities, are the main organs involved in the secretion of royal jelly (Ohashi *et al.*, 1997). The key secretion period occurs when adult honey bees are between 6 and 12 days old. Accordingly, HSP90 and HSP60 may protect the royal jelly protein secretion activity of the hypopharyngeal gland by assisting in the correct folding of proteins, thereby contributing to a high royal jelly production.

## Conclusion

In this study, we measured the production of royal jelly in high royal jelly producing bees and low royal jelly producing bee, putting in evidence a significant difference between the two groups. A total of 369 DEGs were identified between high royal jelly producing bees and low royal jelly producing bees using a cDNA microarray. These DEGs are involved in 46 pathways. This is first comprehensive transcriptome database revealing genes that are differentially expressed between high royal jelly bees and low royal jelly bees. Our results provide new insights into the molecular mechanism of royal jelly secretion and also serve as an extensive novel resource for screening molecular markers to accelerate molecular breeding of high royal jelly bees.

## Supplementary material

The following online information is available for this article:

Table S1 : The details of probes in microarray construction.

Table S2 : Standardized data of high and low royal jelly bees.

Table S3 : Primer sequences of the detected genes.

Table S4 : Information of 369 differentially expressed genes screened by gene chip.

Table S5 : KEGG analysis of the differently expressed genes.
