# Supplementary material for: Identification of genes related to high royal jelly production in the honey bee (Apis mellifera) using microarray analysis
Source: Genet Mol Biol. 2017 Oct 2;40(4):781–9. doi: 10.1590/1678-4685-GMB-2017-0013 (PMC5738612; doi:10.1590/1678-4685-GMB-2017-0013)
Supplement: Supplementary file 3 [file 1415-4757-gmb-1678-4685-GMB-2017-0013-Suppl03.pdf]

Suplementary Material to "Identification of genes related to high royal jelly production in the honey bee (*Apis mellifera*) using microarray analysis"

Table S3. Primer pairs for qRT-PCR.

GI	Gene name	Forward	Reverse	Produce size (bp)
20336614	dopamine receptor type D2 (Dop2)	ATCTGTTGGCTACCTTTCTTCG	AACGCTCTACGAAAATCTCTGC	167
67043607	Amt-2-like protein	ATAGGATTTTTGCTGGCTGGT	TACATTTCTTCGCATCCATC	84
110755554	similar to CG8862-PA (LOC551715)	AGTGGTTGGTATCTGGGAAAGT	GTAGGAACTGCTGCCGAAAC	146
149939402	clone hex71 hexamerin (hex71)	AGTATGGCTGGTTGGCTTATTG	CGATGTTGGCTTCTATGTTCC	191
110759535	hypothetical protein LOC726515 (LOC726515)	AGATCCTAAAAATGGAATTGTGC	CCTCCAAGAACAAGCATTTGA	195
110750767	similar to lethal (1) G0168 CG33206-PA，isoform A(LOC411348)	AACATCAGTGCCTTCAAAACG	CTTCGCTCTCTTCCGTTCTTT	213
66520065	similar to SHC-adaptor protein CG3715-PA (LOC412172)	CACCAGTTCCACCTTTACCAA	CAGTTTCTGCCCATTGTTGAT	162
110749006	similar to CG1998-PA, transcript variant 1 (LOC409360)	GGCAAGCACAATGGGATAAA	TGTAAATCCCGCCAAAGAAC	117
110760992	similar to LDLa domain containing chitin binding protein 1 CG8756-PD, im D, transcript variant 1 (LOC551323)	CAACACAAGTCAGAGCACAGG	GCTTGCTTTGTCGCATCTTAC	149
66538119	similar to Multidrug resistance-associated protein 5 (LOC413947)	TAATGGCGGAAATAGAAACTCG	TGCTCCTGTTCGTCCTACAATA	248
