# Supplementary material for: Identification of genes related to high royal jelly production in the honey bee (Apis mellifera) using microarray analysis
Source: Genet Mol Biol. 2017 Oct 2;40(4):781–9. doi: 10.1590/1678-4685-GMB-2017-0013 (PMC5738612; doi:10.1590/1678-4685-GMB-2017-0013)
Supplement: Supplementary file 4 [file 1415-4757-gmb-1678-4685-GMB-2017-0013-Suppl04.pdf]

Suplementary Material to “Identification of genes related to high royal jelly production in the honey bee (*Apis mellifera*) using microarray analysis”

Table S4. Information of 369 differentially expressed genes screened by gene chip

probe_ID	Accessions	avg_HRJB (log2signal)	avg_LRJB (log2signal)	Ratio	Expression change	Target Description
CUST_5397_PI416562355	GI:110760357	4.618	3.351	2.406	Up	*Apis mellifera* nodal modulator 2-like, transcript variant 1 (LOC725048)
CUST_8371_PI416562355	GI:110767541	6.774	4.983	3.461	Up	similar to CG33257-PA (LOC412520)
CUST_9776_PI416562355	GI:226446422	11.033	9.209	3.540	Up	*Apis mellifera* clone HSP90e heat shock protein 90
CUST_4355_PI416562355	GI:110758302	6.028	4.891	2.200	Up	*Apis mellifera* small G protein signaling modulator 3 homolog (LOC411540)
CUST_898_PI416568369	\	3.600	2.064	2.901	Up	\
CUST_1572_PI416568369	\	3.002	1.939	2.090	Up	\
CUST_3918_PI416562355	GI:110757407	9.682	8.659	2.033	Up	*Apis mellifera* paramyosin, long form-like (LOC409787)
CUST_8510_PI416562355	GI:110768094	4.671	3.264	2.652	Up	*Apis mellifera* hypothetical protein LOC412112 (LOC412112)
CUST_2254_PI416562355	GI:110750767	7.662	6.321	2.533	Up	*Apis mellifera* thyroid receptor-interacting protein 11 (LOC411348)
CUST_3287_PI416562355	GI:110756174	7.852	6.710	2.206	Up	*Apis mellifera* vacuolar protein sorting 28 (Vps28)
CUST_778_PI416562355	GI:66520065	5.356	4.160	2.291	Up	*Apis mellifera* SHC-transforming protein 1 (LOC412172)
CUST_7906_PI416562355	GI:110765957	4.178	2.709	2.767	Up	*Apis mellifera* similar to Guanine nucleotide-releasing protein (LOC726901)
CUST_6919_PI416562355	GI:110763356	5.101	3.125	3.934	Up	*Apis mellifera* uncharacterized LOC726155 (LOC726155)

probe_ID	Accessions	avg_HRJB (log2signal)	avg_LRJB (log2signal)	Ratio	Expression change	Target Description
CUST_6572_PI416562355	GI:110762694	7.894	6.760	2.195	Up	*Apis mellifera* probable leucyl-tRNA synthetase, mitochondrial-like, transcript variant 2 (LOC552532)
CUST_6892_PI416562355	GI:110763305	3.855	2.388	2.765	Up	*Apis mellifera* similar to dynein, axonemal, heavy chain 8 (LOC724774), mRNA
CUST_7104_PI416562355	GI:110763681	7.238	6.017	2.330	Up	*Apis mellifera* cuticular protein CPF2 (CPF2)
CUST_6272_PI416562355	GI:110762080	6.144	4.753	2.623	Up	similar to CG2811-PA (LOC550955)
CUST_8661_PI416562355	GI:110768697	8.941	7.322	3.070	Up	*Apis mellifera* similar to ribosomal protein L5 (LOC726004)
CUST_6807_PI416562355	GI:110763160	5.356	4.316	2.057	Up	*Apis mellifera* protein RFT1 homolog (LOC412489)
CUST_9067_PI416562355	GI:110772770	4.064	2.916	2.215	Up	*Apis mellifera* similar to CG2989-PA (LOC726402)
CUST_5871_PI416562355	GI:110761255	5.476	4.122	2.555	Up	*Apis mellifera* similar to CG9776-PA, isoform A (LOC413337)
CUST_7279_PI416562355	GI:110764025	3.561	2.222	2.530	Up	*Apis mellifera* protein kinase D, transcript variant 2 (PKD)
CUST_1503_PI416562355	GI:110748853	7.010	5.538	2.773	Up	*Apis mellifera* protein ABHD11-like (LOC414038)
CUST_3207_PI416562355	GI:110756036	6.082	4.936	2.212	Up	*Apis mellifera* similar to Sucb CG10622-PA, isoform A (LOC725169)
CUST_1504_PI416568369	\	3.702	2.394	2.477	Up	\
CUST_4150_PI416562355	GI:110757840	4.078	2.959	2.172	Up	*Apis mellifera* similar to expanded CG4114-PA (LOC409898)
CUST_7206_PI416562355	GI:110763898	7.278	5.926	2.553	Up	*Apis mellifera* guanine nucleotide-binding protein-like 3 homolog (LOC724161)
CUST_2842_PI416562355	GI:110755310	5.839	4.777	2.088	Up	*Apis mellifera* mitochondrial ubiquitin ligase activator of nfkb 1-like (LOC409295)
CUST_452_PI416568369	\	3.649	2.587	2.088	Up	\
CUST_1586_PI416562355	GI:110749006	6.130	5.039	2.130	Up	similar to CG1998-PA, transcript variant 1 (LOC409360)
CUST_3045_PI416562355	GI:110755733	7.271	6.232	2.054	Up	*Apis mellifera* cleft lip and palate transmembrane protein 1 homolog (LOC727027)
CUST_1545_PI416562355	GI:110748930	4.920	3.645	2.420	Up	*Apis mellifera* WD repeat-containing protein 66-like (LOC413693)
CUST_4820_PI416562355	GI:110759190	10.446	9.217	2.345	Up	*Apis mellifera* myosin heavy chain 1, transcript variant 1 (Mhc1)
CUST_1493_PI416562355	GI:110748835	7.732	6.723	2.013	Up	*Apis mellifera* vacuolar protein sorting-associated protein 37B (LOC724720)
CUST_9160_PI416562355	GI:110773542	3.038	1.592	2.724	Up	*Apis mellifera* similar to kynurenine aminotransferase III isoform 1 (LOC727352)
CUST_6070_PI416562355	GI:110761686	8.238	6.759	2.788	Up	*Apis mellifera* prostaglandin reductase 1-like (LOC411378)

probe_ID	Accessions	avg_HRJB (log2signal)	avg_LRJB (log2signal)	Ratio	Expression change	Target Description
CUST_907_PI416568369	\	3.778	2.653	2.181	Up	\
CUST_430_PI416562355	GI:66501029	6.969	5.919	2.070	Up	*Apis mellifera* actin-related protein 2 (LOC409313)
CUST_6902_PI416562355	GI:110763328	6.781	5.478	2.468	Up	*Apis mellifera* sideroflexin-3 (LOC408539)
CUST_1209_PI416562355	GI:66558844	5.714	4.600	2.164	Up	*Apis mellifera* breast cancer metastasis-suppressor 1-like protein (LOC552686)
CUST_1464_PI416562355	GI:20336614	4.057	2.892	2.243	Up	*Apis mellifera* dopamine receptor type D2 (Dop2)
CUST_9588_PI416562355	GI:110778049	6.182	5.139	2.061	Up	*Apis mellifera* hedgehog-like (LOC726929)
CUST_906_PI416568369	\	4.045	2.736	2.476	Up	\
CUST_840_PI416562355	GI:66523499	7.660	6.187	2.776	Up	*Apis mellifera* aminomethyltransferase, mitochondrial-like (LOC410550)
CUST_1362_PI416562355	GI:90194187	7.498	5.853	3.129	Up	*Apis mellifera* OBP14
CUST_3690_PI416562355	GI:110756945	3.531	2.001	2.888	Up	*Apis mellifera* solute carrier family 35 member G1-like (LOC724388)
CUST_6444_PI416562355	GI:110762426	3.718	2.393	2.505	Up	similar to CG14446-PA (LOC408403)
CUST_9545_PI416562355	GI:110777694	6.744	5.389	2.557	Up	*Apis mellifera* apolipoprotein D (LOC409278)
CUST_1708_PI416562355	GI:110749242	8.655	7.379	2.421	Up	*Apis mellifera* COX10 homolog, cytochrome c oxidase assembly protein (Cox10)
CUST_2230_PI416562355	GI:110750712	3.936	2.745	2.284	Up	*Apis mellifera* homer protein homolog 2 (LOC413278)
CUST_8919_PI416562355	GI:110771413	3.574	2.497	2.109	Up	*Apis mellifera* similar to Axin (Axis inhibition protein) (dAxin) (d-Axin) (LOC727586)
CUST_8758_PI416562355	GI:110769764	4.795	3.571	2.336	Up	*Apis mellifera* similar to SMC6 protein (LOC727559)
CUST_1453_PI416568369	\	4.188	2.606	2.994	Up	\
CUST_670_PI416568369	\	3.391	2.387	2.006	Up	\
CUST_6241_PI416562355	GI:110762029	7.811	6.446	2.577	Up	*Apis mellifera* endoplasmic reticulum resident protein 44 (LOC552191)
CUST_8096_PI416562355	GI:110766478	3.464	2.112	2.552	Up	similar to ADP/ATP translocase 1 (LOC726168);
CUST_1249_PI416562355	GI:66563289	9.561	8.123	2.709	Up	*Apis mellifera* T-complex protein 1 subunit gamma (LOC409296)
CUST_491_PI416562355	GI:66506313	7.119	6.040	2.113	Up	*Apis mellifera* similar to Probable UDP-glucose 4-epimerase (Galactowaldenase) (LOC411633)

probe_ID	Accessions	avg_HRJB (log2signal)	avg_LRJB (log2signal)	Ratio	Expression change	Target Description
CUST_5718_PI416562355	GI:110760992	7.699	6.517	2.268	Up	*Apis mellifera* uncharacterized LOC551323 (LOC551323)
CUST_6235_PI416562355	GI:110762019	6.507	5.445	2.088	Up	*Apis mellifera* mitochondrial 2-oxoglutarate/malate carrier protein-like (LOC552016)
CUST_8541_PI416562355	GI:110768200	7.345	6.310	2.050	Up	*Apis mellifera* solute carrier family 25 member 40-like, transcript variant 1 (LOC551301)
CUST_8132_PI416562355	GI:110766565	6.390	5.183	2.308	Up	*Apis mellifera* jumonji, AT rich interactive domain 2 (Jarid2)
CUST_1104_PI416562355	GI:66550504	5.095	4.065	2.042	Up	*Apis mellifera* cytochrome c oxidase assembly factor 7 homolog (LOC552586)
CUST_1844_PI416562355	GI:110749527	3.762	2.508	2.385	Up	*Apis mellifera* integrin alpha-8-like (LOC725946)
CUST_5570_PI416562355	GI:110760713	5.075	3.889	2.275	Up	*Apis mellifera* DISCO interacting protein 2 (DIP2)
CUST_9755_PI416562355	GI:218749840	7.644	6.277	2.580	Up	*Apis mellifera* uncharacterized LOC551408 (LOC551408)
CUST_965_PI416562355	GI:66532762	3.816	2.796	2.028	Up	*Apis mellifera* tachykinin-like peptides receptor 99D (LOC411611)
CUST_7858_PI416562355	GI:110765844	7.164	6.049	2.166	Up	*Apis mellifera* protein polybromo-1 (LOC552677)
CUST_9152_PI416562355	GI:110773461	4.763	3.712	2.072	Up	*Apis mellifera* LAG1 longevity assurance homolog 2-like (LOC727082)
CUST_1009_PI416562355	GI:66538119	6.496	5.463	2.046	Up	*Apis mellifera* ATP-binding cassette, sub-family C (CFTR/MRP), member 5 (ABCC5)
CUST_678_PI416562355	GI:66515675	3.726	2.717	2.012	Up	*Apis mellifera* serine/threonine/tyrosine-interacting protein-like (LOC411320)
CUST_3535_PI416562355	GI:110756674	3.896	2.842	2.077	Up	*Apis mellifera* armadillo repeat-containing protein 7-like (LOC412188)
CUST_4271_PI416562355	GI:110758136	3.140	2.016	2.179	Up	*Apis mellifera* hypothetical protein LOC725019 (LOC725019)
CUST_9355_PI416562355	GI:110775710	5.846	4.730	2.167	Up	*Apis mellifera* transmembrane protein 35 (LOC551755)
CUST_9356_PI416562355	GI:110775718	4.008	2.103	3.744	Up	*Apis mellifera* similar to carbonic anhydrase II (LOC727217)
CUST_232_PI416568369	\	3.869	2.551	2.492	Up	\
CUST_6634_PI416562355	GI:110762803	5.591	4.297	2.452	Up	*Apis mellifera* synaptic vesicle glycoprotein 2B-like (LOC412941)
CUST_5738_PI416562355	GI:110761027	5.278	4.221	2.080	Up	*Apis mellifera* similar to CG32705-PA (LOC551683)
CUST_70_PI416562355	GI:18140744	9.819	8.802	2.024	Up	*Apis mellifera* odorant binding protein 4
CUST_5813_PI416562355	GI:110761156	3.867	2.772	2.137	Up	*Apis mellifera* similar to CG13136-PA (LOC724726)
CUST_1392_PI416568369	\	3.305	1.922	2.608	Up	\

probe_ID	Accessions	avg_HRJB (log2signal)	avg_LRJB (log2signal)	Ratio	Expression change	Target Description
CUST_4219_PI416562355	GI:110758023	8.223	7.148	2.107	Up	*Apis mellifera* proteasome 25kD subunit (Pros25)
CUST_6419_PI416562355	GI:110762356	5.203	3.997	2.308	Up	*Apis mellifera* long-chain-fatty-acid--CoA ligase 1-like (LOC412541)
CUST_3369_PI416562355	GI:110756310	9.031	7.930	2.145	Up	*Apis mellifera* ribosomal protein L40 (RpL40)
CUST_5031_PI416562355	GI:110759633	6.509	5.014	2.819	Up	*Apis mellifera* hypothetical protein LOC551133 (LOC551133)
CUST_123_PI416562355	GI:34101242	4.561	3.340	2.331	Up	*Apis mellifera* complementary sex determiner (csd)
CUST_279_PI416568369	\	3.467	2.372	2.136	Up	\
CUST_511_PI416568369	\	3.828	2.660	2.247	Up	\
CUST_1647_PI416562355	GI:110749120	3.699	2.616	2.118	Up	*Apis mellifera* similar to CG9514-PA (LOC724116)
CUST_8653_PI416562355	GI:110768669	3.442	2.213	2.344	Up	*Apis mellifera* similar to CG10252-PA (LOC725443)
CUST_454_PI416568369	\	10.371	9.364	2.010	Up	\
CUST_7955_PI416562355	GI:110766100	4.894	3.802	2.132	Up	*Apis mellifera* similar to CG33722-PC, isoform C (LOC727144)
CUST_5402_PI416562355	GI:110760365	7.673	6.396	2.424	Up	*Apis mellifera* hypothetical protein LOC408285, transcript variant 1 (LOC408285)
CUST_737_PI416568369	\	3.838	2.534	2.468	Up	\
CUST_1062_PI416568369	\	3.558	2.495	2.090	Up	\
CUST_856_PI416568369	\	3.680	2.455	2.338	Up	\
CUST_806_PI416568369	\	4.033	3.027	2.009	Up	\
CUST_333_PI416568369	\	3.972	2.245	3.309	Up	\
CUST_3522_PI416562355	GI:110756648	6.632	5.424	2.310	Up	*Apis mellifera* 40S ribosomal protein S29-like (LOC725147)
CUST_8054_PI416562355	GI:110766354	7.543	6.405	2.202	Up	*Apis mellifera* similar to CG14482-PA (LOC726777)
CUST_8741_PI416562355	GI:110769534	5.627	4.541	2.122	Up	*Apis mellifera* hypothetical protein LOC727095 (LOC727095)
CUST_989_PI416568369	\	3.656	2.501	2.226	Up	\
CUST_4087_PI416562355	GI:110757718	5.065	3.987	2.112	Up	*Apis mellifera* cGMP-dependent 3',5'-cyclic phosphodiesterase-like (LOC412573)
CUST_6428_PI416562355	GI:110762371	10.384	9.294	2.127	Up	*Apis mellifera* cytochrome P450 6AS11 (CYP6AS11)
CUST_4502_PI416562355	GI:110758627	10.368	9.272	2.138	Up	*Apis mellifera* transmembrane emp24 domain-containing protein bai (LOC409026)

probe_ID	Accessions	avg_HRJB (log2signal)	avg_LRJB (log2signal)	Ratio	Expression change	Target Description
CUST_3279_PI416562355	GI:110756160	4.157	2.761	2.632	Up	similar to bcn92 CG3717-PA (LOC725865)
CUST_5512_PI416562355	GI:110760603	6.968	5.841	2.184	Up	*Apis mellifera* ribosomal L1 domain-containing protein 1-like (LOC551532)
CUST_4771_PI416562355	GI:110759097	5.346	3.157	4.559	Up	*Apis mellifera* uncharacterized LOC725454 (LOC725454)
CUST_2707_PI416562355	GI:110755076	4.386	3.255	2.191	Up	*Apis mellifera* Myb-interacting protein 40 (mip40)
CUST_6115_PI416562355	GI:110761760	7.949	6.795	2.226	Up	*Apis mellifera* similar to interferon gamma inducible protein 30 (LOC726203)
CUST_1500_PI416568369	\	3.071	1.888	2.270	Up	\
CUST_4188_PI416562355	GI:110757965	4.042	2.902	2.204	Up	*Apis mellifera* jerky protein homolog-like (LOC724817)
CUST_1213_PI416568369	\	3.351	2.341	2.014	Up	\
CUST_1618_PI416568369	\	3.811	2.803	2.011	Up	\
CUST_9786_PI416562355	GI:229892247	11.811	10.598	2.319	Up	*Apis mellifera* heat shock protein 90 (Hsp90)
CUST_3945_PI416562355	GI:110757459	12.364	11.306	2.081	Up	*Apis mellifera* polyubiquitin-A (LOC409675), transcript variant X1
CUST_1023_PI416562355	GI:66541235	3.836	2.777	2.083	Up	*Apis mellifera* ionotropic receptor 93a-like protein (LOC551697)
CUST_9_PI416562355	GI:433530	10.381	9.171	2.314	Up	major royal jelly protein 4 [*Apis mellifera*]
CUST_1228_PI416568369	\	3.500	2.412	2.126	Up	\
CUST_5547_PI416562355	GI:110760674	6.806	5.806	2.000	Up	*Apis mellifera* uncharacterized LOC724335 (LOC724335)
CUST_6922_PI416562355	GI:110763362	3.475	2.378	2.139	Up	*Apis mellifera* uncharacterized LOC726229 (LOC726229)
CUST_7909_PI416562355	GI:110765966	6.500	5.329	2.251	Up	*Apis mellifera* sec1 family domain-containing protein 2-like (LOC552681)
CUST_7298_PI416562355	GI:110764060	5.337	4.171	2.244	Up	*Apis mellifera* protein FAM192A-like (LOC726096)
CUST_347_PI416568369	\	3.783	2.205	2.985	Up	\
CUST_3513_PI416562355	GI:110756631	4.215	2.899	2.490	Up	*Apis mellifera* kinesin 3D, transcript variant 2 (kinesin-3D)
CUST_5970_PI416562355	GI:110761453	3.735	2.728	2.010	Up	*Apis mellifera* similar to Odorant receptor 24a CG11767-PA (LOC727671)
CUST_1485_PI416568369	\	3.750	2.605	2.211	Up	\
CUST_4124_PI416562355	GI:110757783	3.686	2.584	2.147	Up	*Apis mellifera* hypothetical protein LOC725513 (LOC725513)

probe_ID	Accessions	avg_HRJB (log2signal)	avg_LRJB (log2signal)	Ratio	Expression change	Target Description
CUST_1136_PI416568369	\	4.219	3.016	2.301	Up	\
CUST_9134_PI416562355	GI:110773276	6.976	5.945	2.044	Up	*Apis mellifera* e3 ubiquitin-protein ligase TRIM23-like (LOC409166)
CUST_3851_PI416562355	GI:110757223	3.181	2.072	2.156	Up	*Apis mellifera* iroquois-class homeodomain protein IRX-6-like (LOC726999)
CUST_3591_PI416562355	GI:110756771	4.404	2.706	3.244	Up	*Apis mellifera* septin-7 (LOC725834), transcript variant X1
CUST_8441_PI416562355	GI:110767812	11.449	10.323	2.184	Up	*Apis mellifera* probable Bax inhibitor 1 (LOC552282)
CUST_904_PI416562355	GI:66529172	5.360	3.886	2.779	Up	*Apis mellifera* sister chromatid cohesion protein PDS5 homolog B (LOC551462)
CUST_7176_PI416562355	GI:110763844	8.725	7.659	2.094	Up	*Apis mellifera* 60 kDa heat shock protein, mitochondrial-like (LOC409384)
CUST_79_PI416562355	GI:21321073	7.139	6.108	2.043	Up	*Apis mellifera* Mb-62 mRNA
CUST_850_PI416568369	\	3.435	2.330	2.151	Up	\
CUST_2366_PI416562355	GI:110750980	3.412	2.339	2.104	Up	*Apis mellifera* odorant receptor 22 (Or22)
CUST_1461_PI416562355	GI:110748773	3.931	2.736	2.290	Up	*Apis mellifera* pre-mRNA-splicing factor CWC22 homolog (LOC724331)
CUST_1425_PI416568369	\	3.966	2.532	2.703	Up	\
CUST_1287_PI416562355	GI:67043607	4.135	2.945	2.281	Up	*Apis mellifera* Amt-2-like protein
CUST_2641_PI416562355	GI:110754958	5.132	3.618	2.856	Up	*Apis mellifera* thymidylate kinase (LOC552148)
CUST_6813_PI416562355	GI:110763171	3.930	2.719	2.315	Up	*Apis mellifera* hypothetical protein LOC409090 (LOC409090)
CUST_677_PI416562355	GI:66515668	4.423	3.343	2.115	Up	*Apis mellifera* CG1567-like (LOC411317)
CUST_5014_PI416562355	GI:110759599	4.516	3.134	2.605	Up	*Apis mellifera* neuroligin 1 (NLG-1)
CUST_3700_PI416562355	GI:110756960	11.043	9.955	2.126	Up	*Apis mellifera* glucose dehydrogenase [FAD, quinone] (LOC551044)
CUST_2338_PI416562355	GI:110750931	4.305	2.713	3.016	Up	*Apis mellifera* striatin-interacting protein 1 (LOC412331)
CUST_4776_PI416562355	GI:110759106	8.782	7.419	2.574	Up	*Apis mellifera* hypothetical LOC552685 (LOC552685)
CUST_609_PI416568369	\	3.603	2.486	2.169	Up	\
CUST_4385_PI416562355	GI:110758358	4.158	2.819	2.530	Up	*Apis mellifera* forkhead box protein K2-like (LOC726050)
CUST_917_PI416568369	\	9.852	8.708	2.211	Up	\
CUST_2940_PI416562355	GI:110755490	4.347	2.989	2.564	Up	*Apis mellifera* similar to adrift CG5032-PA (LOC413775)

probe_ID	Accessions	avg_HRJB (log2signal)	avg_LRJB (log2signal)	Ratio	Expression change	Target Description
CUST_971_PI416568369	\	4.147	3.037	2.159	Up	\
CUST_1033_PI416568369	\	3.721	2.712	2.012	Up	\
CUST_7695_PI416562355	GI:110764948	4.224	3.058	2.245	Up	*Apis mellifera* similar to boule CG4760-PB, isoform B (LOC726917)
CUST_3919_PI416562355	GI:110757409	4.026	2.706	2.495	Up	*Apis mellifera* uncharacterized protein KIAA0513 (LOC410135)
CUST_1259_PI416568369	\	3.162	2.135	2.038	Up	\
CUST_1621_PI416568369	\	3.625	2.623	2.003	Up	\
CUST_765_PI416568369	\	3.945	2.666	2.427	Up	\
CUST_3390_PI416562355	GI:110756407	4.391	3.285	2.153	Up	*Apis mellifera* DC-STAMP domain-containing protein 1-like (LOC724561)
CUST_9651_PI416562355	GI:149939402	8.027	6.866	2.236	Up	*Apis mellifera* clone hex71 hexamerin
CUST_2551_PI416562355	GI:110751300	4.317	3.070	2.374	Up	*Apis mellifera* acyl-CoA synthetase family member 4-like (LOC411766)
CUST_85_PI416568369	\	4.094	2.626	2.767	Up	\
CUST_8941_PI416562355	GI:110771577	3.755	2.630	2.180	Up	*Apis mellifera* gem-associated protein 6-like (LOC725227)
CUST_9381_PI416562355	GI:110776003	3.948	2.890	2.082	Up	*Apis mellifera* similar to Wnt oncogene analog 2 CG1916-PA (LOC727397)
CUST_4889_PI416562355	GI:110759368	6.796	5.794	2.003	Up	*Apis mellifera* aspartyl-tRNA synthetase, mitochondrial-like (LOC725136)
CUST_191_PI416562355	GI:48095000	4.024	3.015	2.012	Up	*Apis mellifera* coiled-coil domain-containing protein 64 homolog (LOC410851)
CUST_1255_PI416568369	\	4.056	2.938	2.171	Up	\
CUST_6223_PI416562355	GI:110761996	6.882	5.625	2.390	Up	*Apis mellifera* pyridoxine/pyridoxamine 5'-phosphate oxidase-like (LOC551533)
CUST_978_PI416562355	GI:66533862	5.738	4.518	2.329	Up	*Apis mellifera* N-sulphoglucosamine sulphohydrolase (LOC550884)
CUST_81_PI416562355	GI:22205093	5.420	4.332	2.125	Up	*Apis mellifera* carnica mRNA for CREB 7 protein (creb gene)
CUST_3730_PI416562355	GI:110757016	5.189	3.956	2.351	Up	*Apis mellifera* pyrroline-5-carboxylate reductase 2 (LOC408859)
CUST_8062_PI416562355	GI:110766381	4.231	3.084	2.214	Up	*Apis mellifera* similar to CG1124-PA (LOC726992)
CUST_5223_PI416562355	GI:110760050	3.893	2.839	2.076	Up	*Apis mellifera* mitochondrial pyruvate carrier 2-like (LOC724499)
CUST_1526_PI416568369	\	3.961	2.565	2.631	Up	\
CUST_208_PI416568369	\	4.365	3.251	2.165	Up	\

probe_ID	Accessions	avg_HRJB (log2signal)	avg_LRJB (log2signal)	Ratio	Expression change	Target Description
CUST_1038_PI416568369	\	3.374	2.202	2.253	Up	\
CUST_6457_PI416562355	GI:110762473	7.129	6.080	2.068	Up	*Apis mellifera* coatomer subunit zeta-1 (LOC550981), transcript variant X4
CUST_1992_PI416562355	GI:110749813	5.543	3.279	4.803	Up	*Apis mellifera* tyrosine-protein kinase-like otk-like (LOC410685)
CUST_4006_PI416562355	GI:110757572	4.638	3.566	2.103	Up	*Apis mellifera* krueppel (Kr)
CUST_2249_PI416562355	GI:110750755	8.889	7.830	2.084	Up	*Apis mellifera* uncharacterized LOC410087 (LOC410087)
CUST_706_PI416568369	\	3.932	2.811	2.176	Up	\
CUST_619_PI416568369	\	4.180	3.132	2.068	Up	\
CUST_4812_PI416562355	GI:110759177	5.432	3.942	2.810	Up	*Apis mellifera* ribosome maturation protein SBDS-like (LOC413196)
CUST_1958_PI416562355	GI:110749749	5.317	3.713	3.039	Up	*Apis mellifera* putative adenosylhomocysteinase 3-like (LOC551762)
CUST_1627_PI416568369	\	3.709	2.664	2.063	Up	\
CUST_6539_PI416562355	GI:110762637	4.627	3.495	2.191	Up	*Apis mellifera* small wing (sl)
CUST_1567_PI416562355	GI:110748972	8.585	7.485	2.144	Up	*Apis mellifera* FK506-binding protein FKBP59 (FKBP59)
CUST_7245_PI416562355	GI:110763966	6.919	5.919	2.001	Up	*Apis mellifera* uncharacterized LOC409514 (LOC409514)
CUST_9785_PI416562355	GI:229892213	10.480	9.129	2.551	Up	*Apis mellifera* heat shock 70 kDa protein cognate 3 (Hsc70-3)
CUST_7890_PI416562355	GI:110765921	9.127	8.089	2.053	Up	*Apis mellifera* hypothetical protein LOC727504 (LOC727504)
CUST_162_PI416568369	\	3.686	2.594	2.132	Up	\
CUST_3659_PI416562355	GI:110756888	4.449	3.431	2.026	Up	*Apis mellifera* Wnt11 protein (Wnt11)
CUST_4522_PI416562355	GI:110758661	6.009	4.831	2.262	Up	*Apis mellifera* nucleolar protein 10 (LOC411240)
CUST_8076_PI416562355	GI:110766418	5.682	4.665	2.023	Up	*Apis mellifera* similar to CG5155-PA (LOC412162)
CUST_74_PI416568369	\	3.618	2.574	2.062	Up	\
CUST_3307_PI416562355	GI:110756207	7.018	5.025	3.980	Up	*Apis mellifera* coiled-coil domain-containing protein 123, mitochondrial-like (LOC726602)
CUST_4645_PI416562355	GI:110758878	3.828	2.820	2.011	Up	*Apis mellifera* basement membrane-specific heparan sulfate proteoglycan core

probe_ID	Accessions	avg_HRJB (log2signal)	avg_LRJB (log2signal)	Ratio	Expression change	Target Description
						protein-like (LOC409722)
CUST_1555_PI416568369	\	4.522	3.356	2.243	Up	\
CUST_203_PI416562355	GI:48095664	5.455	3.348	4.309	Up	*Apis mellifera* similar to Ptx1 CG1447-PA, isoform A (LOC411029)
CUST_8065_PI416562355	GI:110766386	4.541	3.208	2.520	Up	*Apis mellifera* protein takeout-like (LOC727021)
CUST_1866_PI416562355	GI:110749567	5.594	6.785	0.438	Down	*Apis mellifera* similar to CG31613-PA (LOC726542)
CUST_1612_PI416568369	\	2.251	3.520	0.415	Down	\
CUST_2974_PI416562355	GI:110755554	3.880	4.897	0.494	Down	*Apis mellifera* endonuclease G, mitochondrial-like (LOC551715)
CUST_73_PI416562355	GI:18265364	7.068	8.072	0.499	Down	*Apis mellifera* Mb-113 mRNA
CUST_8769_PI416562355	GI:110769807	3.778	4.857	0.474	Down	*Apis mellifera* argininosuccinate lyase (LOC551670)
CUST_4981_PI416562355	GI:110759535	4.499	5.772	0.414	Down	*Apis mellifera* uncharacterized protein C11orf70 homolog (LOC726515)
CUST_822_PI416568369	\	2.853	4.045	0.438	Down	\
CUST_7983_PI416562355	GI:110766173	7.519	8.744	0.428	Down	*Apis mellifera* 4-coumarate--CoA ligase 1 (LOC726362), transcript variant X1
CUST_578_PI416562355	GI:66511274	2.791	4.025	0.425	Down	*Apis mellifera* protein mesh (LOC408878)
CUST_2671_PI416562355	GI:110755015	5.747	6.989	0.423	Down	*Apis mellifera* WD repeat-containing protein 46-like (LOC724809), transcript variant X1
CUST_371_PI416562355	GI:66498986	3.970	5.468	0.354	Down	*Apis mellifera* TATA-box-binding protein-like (LOC410753)
CUST_9235_PI416562355	GI:110774378	4.355	5.963	0.328	Down	*Apis mellifera* similar to CG10184-PA (LOC552565)
CUST_289_PI416562355	GI:48139532	3.462	4.514	0.482	Down	*Apis mellifera* serine/threonine-protein kinase 32B-like (LOC413577)
CUST_8605_PI416562355	GI:110768429	2.259	3.684	0.373	Down	*Apis mellifera* hypothetical protein LOC727058 (LOC727058)
CUST_1588_PI416568369	\	2.969	3.996	0.491	Down	\
CUST_7570_PI416562355	GI:110764666	7.777	9.058	0.411	Down	*Apis mellifera* uncharacterized LOC552612 (LOC552612)
CUST_8752_PI416562355	GI:110769673	6.247	7.442	0.437	Down	*Apis mellifera* choline/ethanolamine kinase-like (LOC411525)
CUST_9313_PI416562355	GI:110775273	2.630	3.837	0.433	Down	*Apis mellifera* pantothenate synthetase-like (LOC727315)
CUST_6089_PI416562355	GI:110761718	4.738	6.006	0.415	Down	*Apis mellifera* hypothetical protein LOC725157 (LOC725157)

probe_ID	Accessions	avg_HRJB (log2signal)	avg_LRJB (log2signal)	Ratio	Expression change	Target Description
CUST_5748_PI416562355	GI:110761045	7.219	8.227	0.497	Down	*Apis mellifera* chitinase domain-containing protein 1-like (LOC552683)
CUST_9216_PI416562355	GI:110774174	3.169	4.651	0.358	Down	*Apis mellifera* similar to SMC1 CG6057-PA (LOC727318)
CUST_8357_PI416562355	GI:110767469	5.151	6.336	0.440	Down	*Apis mellifera* similar to CG17999-PA (LOC726751)
CUST_190_PI416562355	GI:48094802	4.674	5.684	0.497	Down	*Apis mellifera* PHD finger protein 2-like (LOC410796)
CUST_3720_PI416562355	GI:110756996	3.895	4.933	0.487	Down	*Apis mellifera* UPF0693 protein C10orf32 homolog (LOC725774)
CUST_1278_PI416562355	GI:66565820	3.527	4.674	0.451	Down	*Apis mellifera* similar to mindbomb homolog 1 (LOC412338)
CUST_5937_PI416562355	GI:110761372	3.084	4.090	0.498	Down	*Apis mellifera* similar to parathyroid hormone-responsive B1 gene (LOC726019)
CUST_6141_PI416562355	GI:110761805	5.948	7.276	0.398	Down	*Apis mellifera* peroxiredoxin-6 (LOC411852)
CUST_2053_PI416562355	GI:110749918	2.236	3.660	0.373	Down	*Apis mellifera* latrophilin Cirl-like (LOC552142)
CUST_2507_PI416562355	GI:110751228	4.832	6.162	0.398	Down	*Apis mellifera* similar to CG17758-PA (LOC410820)
CUST_6722_PI416562355	GI:110762963	3.204	4.358	0.449	Down	*Apis mellifera* tetratricopeptide repeat protein 12-like (LOC727151)
CUST_2404_PI416562355	GI:110751051	4.505	5.784	0.412	Down	*Apis mellifera* RING-box protein 1A (Roc1a)
CUST_3346_PI416562355	GI:110756274	5.268	6.398	0.457	Down	*Apis mellifera* similar to CG14145-PA (LOC725063)
CUST_2124_PI416562355	GI:110750096	3.805	4.928	0.459	Down	*Apis mellifera* hypothetical protein LOC725716 (LOC725716)
CUST_1157_PI416568369	\	1.945	3.574	0.323	Down	\
CUST_8499_PI416562355	GI:110768055	5.426	7.288	0.275	Down	*Apis mellifera* acyl-CoA Delta(11) desaturase (LOC412166)
CUST_1359_PI416562355	GI:90194181	4.394	5.561	0.445	Down	*Apis mellifera* OBP11
CUST_8539_PI416562355	GI:110768198	9.625	11.215	0.332	Down	*Apis mellifera* ATP synthase lipid-binding protein, mitochondrial-like, transcript variant 1 (LOC409148)
CUST_1318_PI416568369	\	3.933	6.171	0.212	Down	\
CUST_893_PI416562355	GI:66526280	4.648	5.817	0.445	Down	*Apis mellifera* scavenger receptor class B member 1 (LOC413408)
CUST_1560_PI416568369	\	2.838	4.027	0.439	Down	\
CUST_8388_PI416562355	GI:110767591	4.901	5.973	0.476	Down	*Apis mellifera* CMP-sialic acid transporter 1 (LOC552387)
CUST_779_PI416568369	\	2.884	3.947	0.479	Down	\

probe_ID	Accessions	avg_HRJB (log2signal)	avg_LRJB (log2signal)	Ratio	Expression change	Target Description
CUST_930_PI416568369	\	2.754	3.785	0.490	Down	\
CUST_6363_PI416562355	GI:110762248	2.976	3.997	0.493	Down	*Apis mellifera* coiled-coil domain-containing protein 113 (LOC726613)
CUST_4332_PI416562355	GI:110758258	3.197	4.446	0.421	Down	*Apis mellifera* aminopeptidase N-like (LOC551180)
CUST_515_PI416568369	\	2.400	3.509	0.464	Down	\
CUST_3016_PI416562355	GI:110755682	2.766	3.850	0.472	Down	*Apis mellifera* transcriptional activator cubitus interruptus (LOC551746)
CUST_6846_PI416562355	GI:110763230	4.578	5.803	0.428	Down	*Apis mellifera* similar to U1 small nuclear ribonucleoprotein A (LOC409948)
CUST_2412_PI416562355	GI:110751064	2.711	3.909	0.436	Down	*Apis mellifera* LIM domain only protein 3-like (LOC724851)
CUST_843_PI416568369	\	2.595	3.604	0.497	Down	\
CUST_6335_PI416562355	GI:110762198	4.302	5.345	0.485	Down	*Apis mellifera* hemicentin-2-like (LOC725619)
CUST_823_PI416568369	\	2.926	3.956	0.490	Down	\
CUST_615_PI416568369	\	2.928	3.975	0.484	Down	\
CUST_8257_PI416562355	GI:110767159	2.392	3.559	0.445	Down	*Apis mellifera* similar to CG5278-PA (LOC727002)
CUST_2899_PI416562355	GI:110755411	2.713	3.813	0.466	Down	*Apis mellifera* similar to CG7466-PA (LOC725863)
CUST_6350_PI416562355	GI:110762224	3.319	4.372	0.482	Down	*Apis mellifera* similar to Ca2+-channel-protein--subunit CG6320-PD, isoform D, (LOC408430)
CUST_3108_PI416562355	GI:110755849	7.141	8.197	0.481	Down	*Apis mellifera* myosin 18 (Myo18)
CUST_510_PI416568369	\	2.645	3.835	0.438	Down	\
CUST_3452_PI416562355	GI:110756526	2.744	3.959	0.431	Down	*Apis mellifera* cilia- and flagella-associated protein 57 (LOC411073)
CUST_9030_PI416562355	GI:110772423	5.160	6.274	0.462	Down	*Apis mellifera* similar to rab3-GEF CG5627-PA (LOC727374)
CUST_378_PI416568369	\	2.753	4.268	0.350	Down	\
CUST_1673_PI416568369	\	5.205	6.388	0.440	Down	\
CUST_7636_PI416562355	GI:110764820	5.055	6.301	0.422	Down	*Apis mellifera* peroxisomal membrane protein PEX13-like (LOC727257)
CUST_4151_PI416562355	GI:110757849	2.736	4.306	0.337	Down	*Apis mellifera* similar to adenylate kinase 7 (LOC411160)
CUST_854_PI416562355	GI:66524123	2.410	3.801	0.381	Down	*Apis mellifera* carboxypeptidase B-like (LOC551327)

probe_ID	Accessions	avg_HRJB (log2signal)	avg_LRJB (log2signal)	Ratio	Expression change	Target Description
CUST_183_PI416562355	GI:47157063	5.151	6.448	0.407	Down	*Apis mellifera* ligustica ubiquinone biosynthesis protein COQ7-like protein (clk-1)
CUST_6101_PI416562355	GI:110761737	3.189	4.289	0.467	Down	*Apis mellifera* leucine-rich repeat-containing protein 15 (LOC410373)
CUST_3382_PI416562355	GI:110756385	4.821	5.986	0.446	Down	*Apis mellifera* zinc finger protein 816-like (LOC724513)
CUST_1563_PI416568369	\	2.327	3.413	0.471	Down	\
CUST_2919_PI416562355	GI:110755446	6.429	7.444	0.495	Down	*Apis mellifera* succinate dehydrogenase [ubiquinone] flavoprotein subunit (LOC408734)
CUST_350_PI416568369	\	2.730	3.755	0.492	Down	\
CUST_818_PI416562355	GI:66522186	3.305	4.584	0.412	Down	*Apis mellifera* beta-1,3-galactosyltransferase 1-like (LOC552398)
CUST_3023_PI416562355	GI:110755693	5.053	6.406	0.392	Down	*Apis mellifera* ADAM 17-like protease (LOC551599)
CUST_6838_PI416562355	GI:110763216	3.362	4.569	0.433	Down	*Apis mellifera* ATP-dependent DNA helicase Q5-like (LOC725113)
CUST_98_PI416568369	\	2.193	3.222	0.490	Down	\
CUST_2264_PI416562355	GI:110750794	4.807	6.408	0.330	Down	*Apis mellifera* arrestin domain-containing protein 4 (LOC410896)
CUST_4001_PI416562355	GI:110757561	4.933	5.966	0.489	Down	*Apis mellifera* n-acetyltransferase 10-like (LOC724656)
CUST_772_PI416568369	\	2.634	3.980	0.393	Down	\
CUST_4173_PI416562355	GI:110757935	2.472	3.640	0.445	Down	*Apis mellifera* peroxidase (Pxd)
CUST_6512_PI416562355	GI:110762589	4.021	5.066	0.485	Down	*Apis mellifera* similar to Transcriptional regulator ATRX homolog (LOC724593)
CUST_2640_PI416562355	GI:110754955	3.294	4.300	0.498	Down	*Apis mellifera* hypothetical protein LOC724650 (LOC724650)
CUST_2327_PI416562355	GI:110750909	2.424	3.920	0.355	Down	*Apis mellifera* hypothetical protein LOC410882, transcript variant 1 (LOC410882)
CUST_8951_PI416562355	GI:110771666	2.136	3.501	0.388	Down	*Apis mellifera* hypothetical protein LOC726809 (LOC726809)
CUST_8372_PI416562355	GI:110767542	6.067	7.427	0.390	Down	*Apis mellifera* similar to Probable cytochrome P450 6a14 (CYPVIA14) (LOC552418)
CUST_137_PI416568369	\	1.608	3.092	0.358	Down	\
CUST_1266_PI416562355	GI:66564902	3.268	4.382	0.462	Down	*Apis mellifera* serine/threonine-protein kinase SIK2 (LOC413736)
CUST_8027_PI416562355	GI:110766294	3.827	4.987	0.448	Down	*Apis mellifera* lachesin-like (LOC725840)

probe_ID	Accessions	avg_HRJB (log2signal)	avg_LRJB (log2signal)	Ratio	Expression change	Target Description
CUST_8361_PI416562355	GI:110767492	5.120	6.190	0.476	Down	*Apis mellifera* cardioacceleratory peptide receptor (LOC726935)
CUST_8770_PI416562355	GI:110769829	3.838	4.906	0.477	Down	*Apis mellifera* probable cytochrome P450 6a13 (LOC727598)
CUST_461_PI416562355	GI:66504571	3.070	4.231	0.447	Down	*Apis mellifera* similar to CG18265-PA (LOC410861)
CUST_388_PI416568369	\	3.014	4.049	0.488	Down	\
CUST_979_PI416568369	\	2.758	3.877	0.460	Down	\
CUST_817_PI416568369	\	2.267	3.309	0.486	Down	\
CUST_7904_PI416562355	GI:110765951	3.024	4.066	0.486	Down	*Apis mellifera* cytochrome P450 6A1 (LOC413908)
CUST_5017_PI416562355	GI:110759606	2.516	3.663	0.452	Down	*Apis mellifera* hypothetical protein LOC724572 (LOC724572)
CUST_6841_PI416562355	GI:110763221	4.645	5.885	0.423	Down	*Apis mellifera* choline/ethanolaminephosphotransferase 1-like (LOC552795)
CUST_3733_PI416562355	GI:110757021	3.204	4.478	0.413	Down	*Apis mellifera* hypothetical protein LOC726189 (LOC726189)
CUST_8809_PI416562355	GI:110770106	2.664	3.787	0.459	Down	*Apis mellifera* RNA-binding protein 1 (LOC727349)
CUST_1108_PI416568369	\	2.512	3.539	0.491	Down	\
CUST_3153_PI416562355	GI:110755933	7.934	9.146	0.432	Down	*Apis mellifera* uncharacterized LOC408807 (LOC408807)
CUST_6146_PI416562355	GI:110761812	3.078	4.193	0.462	Down	*Apis mellifera* PEST proteolytic signal-containing nuclear protein-like (LOC726782)
CUST_6055_PI416562355	GI:110761656	2.633	3.642	0.497	Down	*Apis mellifera* basic helix-loop-helix neural transcription factor TAP (LOC725085)
CUST_1168_PI416568369	\	2.917	3.921	0.499	Down	\
CUST_5175_PI416562355	GI:110759959	3.372	4.403	0.489	Down	*Apis mellifera* matrix metalloproteinase-14 (LOC409868)
CUST_484_PI416568369	\	2.939	3.948	0.497	Down	\
CUST_537_PI416568369	\	6.321	7.589	0.415	Down	\
CUST_730_PI416568369	\	2.309	3.715	0.377	Down	\
CUST_1229_PI416568369	\	2.012	3.064	0.482	Down	\
CUST_5247_PI416562355	GI:110760094	6.151	7.164	0.495	Down	*Apis mellifera* small nuclear ribonucleoprotein F, transcript variant 1 (DebB)
CUST_847_PI416562355	GI:66523769	2.722	3.781	0.480	Down	*Apis mellifera* uncharacterized oxidoreductase YjmC (LOC410520)
CUST_1262_PI416562355	GI:66564816	6.498	7.572	0.475	Down	*Apis mellifera* methyltransferase-like protein 13 (LOC412624)

probe_ID	Accessions	avg_HRJB (log2signal)	avg_LRJB (log2signal)	Ratio	Expression change	Target Description
CUST_368_PI416568369	\	2.519	3.528	0.497	Down	\
CUST_7101_PI416562355	GI:110763677	9.729	10.948	0.430	Down	*Apis mellifera* ribosomal protein L26, transcript variant 1 (RpL26)
CUST_9126_PI416562355	GI:110773257	2.273	3.451	0.442	Down	*Apis mellifera* similar to glutathione reductase 1 (LOC727640)
CUST_6166_PI416562355	GI:110761845	3.214	4.446	0.426	Down	*Apis mellifera* uncharacterized LOC724959 (LOC724959)
CUST_8854_PI416562355	GI:110770770	2.148	3.156	0.497	Down	*Apis mellifera* hypothetical protein LOC727557 (LOC727557)
CUST_888_PI416568369	\	3.013	4.015	0.499	Down	\
CUST_4055_PI416562355	GI:110757666	2.645	3.654	0.497	Down	*Apis mellifera* similar to ADAM metallopeptidase with thrombospondin type 1 motif, (LOC724351)
CUST_4606_PI416562355	GI:110758815	6.043	7.105	0.479	Down	*Apis mellifera* ubiquinone biosynthesis O-methyltransferase, mitochondrial (LOC412082)
CUST_7050_PI416562355	GI:110763589	6.603	7.680	0.474	Down	*Apis mellifera* similar to grapes CG17161-PA, isoform A, transcript variant 1 (LOC413964)
CUST_3768_PI416562355	GI:110757082	6.021	7.304	0.411	Down	*Apis mellifera* annexin-B9-like (LOC412487)
CUST_3104_PI416562355	GI:110755841	5.053	6.156	0.466	Down	*Apis mellifera* protein FAM114A2 (LOC551672)
CUST_6057_PI416562355	GI:110761660	2.661	4.203	0.343	Down	*Apis mellifera* hypothetical protein LOC725198 (LOC725198)
CUST_7327_PI416562355	GI:110764119	2.113	3.217	0.465	Down	*Apis mellifera* spermine oxidase-like (LOC725007)
CUST_8042_PI416562355	GI:110766327	6.228	7.421	0.437	Down	*Apis mellifera* cleavage and polyadenylation specificity factor subunit CG7185 ortholog (LOC551973)
CUST_8970_PI416562355	GI:110771859	4.747	5.870	0.459	Down	*Apis mellifera* similar to Glycerol-3-phosphate dehydrogenase [NAD+], (LOC727482)
CUST_2509_PI416562355	GI:110751231	2.931	3.961	0.490	Down	*Apis mellifera* similar to CG17761-PA (LOC725010)
CUST_3237_PI416562355	GI:110756085	2.814	3.864	0.483	Down	*Apis mellifera* chameau (chm)
CUST_1085_PI416568369	\	2.628	3.703	0.475	Down	\
CUST_503_PI416568369	\	3.058	4.801	0.299	Down	\
CUST_606_PI416568369	\	2.683	3.748	0.478	Down	\

probe_ID	Accessions	avg_HRJB (log2signal)	avg_LRJB (log2signal)	Ratio	Expression change	Target Description
CUST_119_PI416568369	\	2.948	4.053	0.465	Down	\
CUST_8788_PI416562355	GI:110769967	6.101	7.554	0.365	Down	*Apis mellifera* probable ATP-dependent RNA helicase DDX17-like (LOC726768)
CUST_2655_PI416562355	GI:110754983	2.744	3.762	0.494	Down	*Apis mellifera* uncharacterized LOC725791 (LOC725791)
CUST_7792_PI416562355	GI:110765247	5.516	6.622	0.465	Down	*Apis mellifera* hypothetical protein LOC551908, transcript variant 2 (LOC551908)
CUST_4285_PI416562355	GI:110758165	7.027	8.147	0.460	Down	*Apis mellifera* similar to sallimus CG1915-PC, isoform C (LOC551181)
CUST_9077_PI416562355	GI:110772836	5.130	6.721	0.332	Down	*Apis mellifera* similar to CG5537-PA (LOC727016)
CUST_6087_PI416562355	GI:110761715	3.685	4.933	0.421	Down	*Apis mellifera* hypothetical protein LOC411361 (LOC411361)
CUST_62_PI416562355	GI:12659340	4.045	5.702	0.317	Down	*Apis mellifera* odorant binding protein (OBP-8)
CUST_8103_PI416562355	GI:110766502	5.981	7.123	0.453	Down	*Apis mellifera* renin receptor-like (LOC552228)
CUST_1785_PI416562355	GI:110749395	2.691	3.704	0.496	Down	*Apis mellifera* ecdysis triggering hormone receptor (Ethr)
CUST_9195_PI416562355	GI:110773944	4.260	5.284	0.492	Down	*Apis mellifera* similar to Cholinesterase precursor (LOC727064)
CUST_6075_PI416562355	GI:110761695	9.868	11.045	0.442	Down	*Apis mellifera* ribosomal protein S3A (RpS3A)
CUST_760_PI416562355	GI:66519406	5.158	6.190	0.489	Down	*Apis mellifera* Smad anchor for receptor activation (Sara)
CUST_5229_PI416562355	GI:110760063	6.365	7.369	0.499	Down	*Apis mellifera* similar to SET domain containing 3 (LOC410155)
CUST_9549_PI416562355	GI:110777719	3.092	4.315	0.428	Down	*Apis mellifera* hypothetical protein LOC726008 (LOC726008)
CUST_1750_PI416562355	GI:110749329	6.083	7.210	0.458	Down	*Apis mellifera* 6-phosphogluconolactonase (LOC551785)
CUST_7069_PI416562355	GI:110763621	5.701	6.711	0.496	Down	*Apis mellifera* phosphatidylinositol 4-kinase II beta (Pi4k2b)
CUST_6959_PI416562355	GI:110763430	9.503	10.601	0.467	Down	*Apis mellifera* chymotrypsin inhibitor (LOC725114)
CUST_639_PI416562355	GI:66513917	4.587	5.613	0.491	Down	*Apis mellifera* uncharacterized LOC413618 (LOC413618)
CUST_2251_PI416562355	GI:110750761	6.800	7.867	0.477	Down	*Apis mellifera* similar to Protein lethal(2)essential for life (Protein Efl21) (LOC724405)
CUST_2602_PI416562355	GI:110751392	7.032	8.057	0.491	Down	*Apis mellifera* GPI transamidase component PIG-T (LOC410132)
CUST_1751_PI416562355	GI:110749330	5.751	6.868	0.461	Down	*Apis mellifera* protein ABHD13 (LOC412636)
CUST_12_PI416562355	GI:562091	8.315	9.779	0.363	Down	*Apis mellifera* hymenoptaecin
CUST_5950_PI416562355	GI:110761392	8.007	9.115	0.464	Down	*Apis mellifera* histone deacetylase 5 (LOC408331)

probe_ID	Accessions	avg_HRJB (log2signal)	avg_LRJB (log2signal)	Ratio	Expression change	Target Description
CUST_7730_PI416562355	GI:110765056	5.890	7.041	0.450	Down	*Apis mellifera* pitchoune (pit)
CUST_2738_PI416562355	GI:110755132	6.586	7.956	0.387	Down	*Apis mellifera* ubiquitin-protein ligase E3B-like (LOC408749)
CUST_56_PI416568369	\	6.917	8.027	0.463	Down	\
CUST_7211_PI416562355	GI:110763907	8.471	9.567	0.468	Down	*Apis mellifera* similar to CG16791-PA (LOC408555)
CUST_5629_PI416562355	GI:110760833	8.498	9.917	0.374	Down	*Apis mellifera* 26S protease regulatory subunit 8, transcript variant 1 (LOC550794)
CUST_3248_PI416562355	GI:110756105	6.745	8.330	0.333	Down	*Apis mellifera* scavenger receptor class B, type 9 (SCR-B9), transcript variant X6
CUST_3324_PI416562355	GI:110756237	7.953	8.985	0.489	Down	*Apis mellifera* V-type proton ATPase subunit G (LOC551961)
CUST_3182_PI416562355	GI:110755986	3.592	4.725	0.456	Down	*Apis mellifera* hypothetical protein LOC726023 (LOC726023)
CUST_6505_PI416562355	GI:110762576	3.844	5.170	0.399	Down	*Apis mellifera* hypothetical protein LOC724121, transcript variant 1 (LOC724121)
CUST_13_PI416562355	GI:882407	10.522	12.181	0.317	Down	*Apis mellifera* long-wavelength rhodopsin
CUST_3620_PI416562355	GI:110756821	8.063	9.114	0.483	Down	*Apis mellifera* similar to Protein yippee-like 1 (LOC408838)
CUST_780_PI416562355	GI:66520255	6.170	7.320	0.451	Down	*Apis mellifera* electron transfer flavoprotein subunit beta (LOC410308)
CUST_762_PI416568369	\	3.018	4.144	0.458	Down	\

Note: "\" represent gene, without publishing in NCBI
