# Supplementary material for: Identification of genes related to high royal jelly production in the honey bee (Apis mellifera) using microarray analysis
Source: Genet Mol Biol. 2017 Oct 2;40(4):781–9. doi: 10.1590/1678-4685-GMB-2017-0013 (PMC5738612; doi:10.1590/1678-4685-GMB-2017-0013)
Supplement: Supplementary file 5 [file 1415-4757-gmb-1678-4685-GMB-2017-0013-Suppl05.pdf]

Suplementary Material to "Identification of genes related to high royal jelly production in the honey bee (*Apis mellifera*) using microarray analysis"

Table S5. KEGG analysis of the differently expressed genes in the HRJBs and LRJBs samples

KEGG pathway	description	Associated genes- NCBI ID
Ame 00510	N-Glycan biosynthesis	LOC412489
Ame 04310	Wnt signaling pathway	LOC408691
Ame 00130	Ubiquinone and other terpenoid- quinone biosynthesis	LOC412082
Ame 04070	Phosphatidylinositol signaling system	LOC551709, LOC725812
Ame 00330	Arginine and proline metabolism	LOC551709, LOC725812
Ame 04146	Peroxisome	LOC412541, LOC727257
Ame 03040	Spliceocome	LOC726768, LOC725880
Ame 01040	Biosynthesis of unsaturated fatty acids	LOC412166
Ame 03050	Proteasome	LOC550794, LOC479802
Ame 00260	Glycine, serine and threonine metabolism	LOC552565, LOC410550
Ame 00360	Phenylalanine metabolism	LOC411852
Ame 00860	Porphyrin and chlorophyll metabolism	LOC409103
Ame 00230	Purine metabolism	LOC412573
Ame 00670	One carbon pool by folate	LOC410550
Ame 04144	Endoctosis	LOC413457, LOC408786, LOC724000
Ame 00030	Pentose phosphate pathway	LOC551785
Ame 04340	Hedgehog signaling pathway	LOC551746, LOC726929
Ame 04710	Circadian rhythm-mammal	LOC408691
Ame 00071	Fatty acid metabolism	LOC412541
Ame 04650	Natural killer cell mediated cytotoxicity	LOC551709, LOC412172
Ame 00531	Glycosminoglycan degradation	LOC550884
Ame 03010	Ribosome	LOC413296, LOC408515, LOC725147, LOC725943
Ame 04141	Protein processing in endoplasmic reticulum	LOC408691
Ame 00100	Steroid biosynthesis	LOC409360
Ame 00564	Glycerophospholipid metabolism	LOC411525
Ame 03420	Nucleotide excision repair	LOC408691
Ame 00270	Cysteine and methionine metabolism	LOC551762
Ame 00052	Galactose metabolism	LOC411633
Ame 00520	Amino sugar and nucleotide sugar metabolism	LOC411633

KEGG pathway	description	Associated genes- NCBI ID
Ame 03018	RNA degradation	LOC409384
Ame 00910	Nitrogen metabolism	LOC410550
Ame 00601	Glycosphingolipid biosynthesis-lacto and neolacto series	LOC552398
Ame 00970	Aminoacyl-tRNA biosynthesis	LOC725136
Ame 04350	TGF-beta signaling pathway	LOC413457, LOC408691
Ame 00562	Inositol phosphate metabolism	LOC551709, LOC725812
Ame 03013	RNA transport	LOC725227
Ame 00190	Oxidative phosphorylation	LOC551961, LOC409148, LOC409103, LOC726777
Ame 02010	ABC transporters	LOC413947
Ame 04145	Phagosome	LOC551961
Ame 04142	Lysosome	LOC550884
Ame 04512	ECM-receptor interaction	LOC412941
Ame 00240	Pyrimidine metabolism	LOC552148
Ame 03008	Ribosome biogenesis in eukaryotes	LOC413196, LOC724656, LOC724000
Ame 04080	Neuroactive ligand-receptor interaction	LOC411611
Ame 04120	Ubiquitin mediated proteolysis	LOC408749, LOC408691
Ame 00563	Glycosylphosphatidylinositol(GPI)-anchor biosynthesis	LOC410132
